# Proteomic biomarkers for survival in systemic sclerosis-associated pulmonary hypertension

**DOI:** 10.1186/s12931-023-02578-0

**Published:** 2023-11-07

**Authors:** Valentine Mismetti, Xavier Delavenne, David Montani, Souad Bezzeghoud, Olivier Delezay, Sophie Hodin, David Launay, Sylvain Marchand-Adam, Hilario Nunes, Edouard Ollier, Martine Reynaud-Gaubert, Jean Pastre, Julie Traclet, Sébastien Quetant, Sabrina Zeghmar, Laurent Bertoletti, Vincent Cottin

**Affiliations:** 1 Université Jean Monnet Saint-Étienne, Mines Saint-Etienne, INSERM, SAINBIOSE U1059, 42023 Saint-Etienne, France; 2 Service de Pneumologie et Soins Intensifs Thoraciques, Centre de Référence de L’Hypertension Pulmonaire, INSERM U999 Centre de Compétence Maladies Pulmonaires Rares—OrphaLung Hôpital de Bicêtre, Le Kremlin Bicêtre, Paris, France; 3 Service de Médecine Vasculaire et Thérapeutique, INSERM, CIC-1408, CHU de Saint-Etienne, Saint-Etienne, France; 4 Service de Médecine Interne et d’Immunologie Clinique, Centre de Référence des Maladies Auto-Immunes et Systémiques Rares du Nord et Nord-Ouest de France (CeRAINO) CHU Lille, Lille, France; 5 Service de Pneumologie et Explorations Fonctionnaires Respiratoires, Tours, France; 6 Service de Pneumologie, Centre de Référence des Maladies Pulmonaires Rares, Hôpital Avicenne, INSERM U1272, Université Sorbonne Paris Nord, Bobigny, France; 7 Centre de Compétence des Maladies Pulmonaires Rares (OrphaLung), Service de Pneumologie et Transplantation Pulmonaire, CHU Nord, AP-HM, Aix Marseille Université, Marseille, France; 8 Service de Pneumologie et Soins Intensifs Centre de Compétence Maladie Pulmonaire Rare (OrphaLung) Hôpital Européen Georges Pompidou, AP-HP, Paris, France; 9 National Reference Center for Rare Pulmonary Diseases, Department of Respiratory Diseases, Hospices Civils de Lyon, Louis Pradel Hospital, 69677 Lyon, France; 10 Service de Pneumologie, Grenoble, France; 11 Service de Médecine Vasculaire et Thérapeutique, INSERM, UMR1059, Université Jean-Monnet, INSERM, CIC-1408, CHU de Saint-Etienne, INNOVTE, 42055 Saint-Etienne, France; 12 Univ Lyon, INRA, UMR754, 69008 Lyon, France

**Keywords:** Pulmonary hypertension, Systemic sclerosis, Proteomics analysis, Proteins, Biomarkers, Mass spectrometry

## Abstract

**Background:**

Interstitial lung disease (ILD) and pulmonary hypertension (PH) represent the major causes of mortality in systemic sclerosis (SSc). Patients with systemic sclerosis and combined PH and ILD (SSc-PH-ILD) generally have a poor prognosis. Predictors of survival and of potential benefit of treatment are lacking in patients with SSc-PH-ILD.

**Objective:**

To identify specific plasma protein expression patterns associated with survival in patients with SSc-PH-ILD.

**Materials and methods:**

Post-hoc analysis of a prospective multicenter French study in patients with PH-ILD. An untargeted proteomic analysis using mass spectrometry was performed to identify plasma protein changes associated with long-term overall survival in patients with SSc-PH-ILD.

**Results:**

Thirty two patients were included in the analysis, of whom 13 died during follow-up (median survival: 76.5 months). At baseline, survivors had less severe hemodynamic impairment [pulmonary vascular resistance of 4.4 Wood Units (IQR 3–5.2) vs. 6.2 Wood Units (IQR 4.2–10.7)] and higher carbon monoxide diffusing capacity [median 39% (IQR 35–44%) vs. 25% (IQR 22–30.5%)], than the 13 patients who died. Seven proteins, associated with haemostasis and fibrosis, were differentially expressed according to patients’ survival. In the survivor group, two proteins were increased (ADAMTS13, SERPIND1) and five were decreased (PTGDS, OLFM1, C7, IGFBP7, FBN1) compared to the non-survivor groups.

**Conclusion:**

The prognosis of SSc-PH-ILD patients is poor. This proteomic approach found 7 plasma proteins (involved in haemostasis and fibrosis pathways) associated with survival. These potential biomarkers may be good candidates to prognostic enrichment.

**Supplementary Information:**

The online version contains supplementary material available at 10.1186/s12931-023-02578-0.

## Introduction

Systemic sclerosis (SSc) is a rare and complex autoimmune disease characterized by processes that combine immune-mediated inflammation, vasculopathy and fibrosis [1]. Most organs may be affected, but the fibrotic and vascular pulmonary manifestations, particularly interstitial lung disease (ILD) and pulmonary hypertension (PH), stand as the principal sources of morbidity and mortality [1–3].

Within the context of SSc, the prevalence of PH ranges from 8 to 15% [4–6], and can be included in various classifications. In SSc, the two main categories of PH, are pulmonary arterial hypertension (PAH) (accounting for approximately two-third of patients with SSc and PH) and PH associated to ILD (affecting one-third of patients). In SSc-associated PAH (SSc-PAH, Group 1), progressive pulmonary vascular resistance increase results from pulmonary vascular remodeling due to endothelial dysfunction [7]. The therapies designed for specific PAH target endothelial dysfunction and pulmonary vascular remodeling, leading to decreased pulmonary vascular resistance.

The coexistence of ILD and PH significantly impacts prognosis, with a 3-year overall survival rate of 35%, with a poorer prognosis than in patients with isolated SSc-PAH [8]. Furthermore, the efficacy of PAH therapy in patients with SSc-PH-ILD remains uncertain, and certain compounds raise safety concerns [9].

Classifying SSc patients into group 1 and group 3 PH can be challenging [10]. PH associated with extensive ILD (> 20% extent of fibrosis on High-Resolution Computed Tomography (HRCT) or forced vital capacity (FVC) < 70% in case of indeterminate HRCT) is typically categorized under group 3. However, for a significant number of patients, precise differentiation between the two groups based on these parameters may be challenging [11]. The main issue is to accurately separate group 1 and group 3 in patients with concurrent ILD. In certain instances, a patient classified under group 3 of the classification may also present pathophysiological typically associated with group 1. This uncertainty raises questions about the impact of PAH therapies on the outcome in patients with “mixed” disease.

There is a paucity of data regarding the effectiveness of specific PAH treatments in patients with SSc-PH-ILD. While some studies have indicated that PAH-targeted therapies may improve haemodynamics in SSc-PH-ILD but they do not demonstrate any benefit in dyspnoea and survival and there might even be an elevated risk of hypoxia [12, 13]. The positive hemodynamic response to treatment observed in certain patient cohorts [13] suggests an overlap between SSc-PAH and SSc-PH-ILD. Consequently, a careful phenotyping of PH in SSc is essential, as it can significantly influence the selection of treatment and prognosis [9, 14].

Here, we hypothesized that the biological processes underlying the pathophysiology of SSc-PH-ILD might be reflected by perturbations in circulating proteins. Plasma protein level alterations have been previously employed to investigate the molecular drivers of PAH [15–18]. The aim of this exploratory study was to identify specific plasma protein expression patterns associated with survival in patients with SSc-PH-ILD.

## Material and methods

### Study design

This is a post-hoc analysis with data extracted from a clinical database and biobank already constituted from the Pulmonary Hypertension in Interstitial Lung Disease (HYPID) and HYPID-2 consecutive observational prospective studies (ClinicalTrials.gov identifiers: NCT01443598 and NCT02799771, respectively), which aimed to identify prognostic factors in PH associated with ILD. The HYPID study (NCT01443598) was a prospective multicenter study (15 centres) coordinated by the national reference center for rare pulmonary diseases, Louis Pradel hospital, Lyon (France), which results in nested analysis with the French PH registry have been published [13]. Human biological samples and associated data were obtained from NeuroBioTec (CRB HCL, Lyon France, Biobank BB-0033-00046).

Informed consent was obtained from all patients. The present study was approved by the institutional review board of the Hospices Civils de Lyon (approved December 6, 2017). The Comité National d’Informatique et Liberté (CNIL), the committee dedicated to privacy, information technology, and civil rights in France, approved the reference methodology MR03 that was used to collect and analyze registry data (approval no. 17-215). The HYPID and HYPID-2 studies were approved by the CNIL and the Comité de Protection des Personnes (approved January 5, 2011) and the institutional review board (approval CEPRO 2016-021).

### Patients and follow-up

Patients were eligible for inclusion in this study if they met the following criteria: age ≥ 18 years; diagnosis of SSc according to the American College of Rheumatology/European League Against Rheumatism 2013 criteria [19]; precapillary PH demonstrated by RHC, with a mean pulmonary artery pressure (PAP) of ≥ 25 mm Hg, a mean pulmonary vascular resistance (PVR) of > 3 Wood units, and a mean pulmonary artery wedge pressure (PAWP) of ≤ 15 mmHg according to the ERS/ESC 2015 definition [9, 14]; no evidence of chronic thromboembolic PH; ≥ 1 CT scan of the chest available for review; pulmonary function tests available at baseline; and receipt of ≥ 1 dose of targeted PAH treatment within 3 months after PH diagnosis.

Patients with postcapillary PH or probable pulmonary veno-occlusive disease, according to published criteria [9, 14], were excluded. Since the French PH registry was established before the 2018 revision of the PH definition, PH was defined by a mean PAP of ≥ 25 mm Hg.

All CT scans were thoroughly examined by a pneumologist and a radiologist with expertise in ILD, to assess the presence and extent of ILD [20], and to classify the ILD pattern through consensus. This review was not possible for 3 patients because CT images could not be retrieved, although radiologist reports were available and confirmed ILD.

To confirm the presence of ILD, the most recent available chest CT scan was reviewed. If the most recent CT scan indicated no evidence of ILD, patients were classified as SSc-PAH (and consequently excluded from our study). If it showed evidence of ILD, the CT scan obtained at the time of PH diagnosis was examined. In systemic sclerosis, differentiating group 1 PAH from group 3 ILD-PH may be challenging. Therefore, if the CT scan already showed ILD at the time of PH diagnosis, patients were classified as SSc-PH-ILD, irrespective of the physiological alteration and extent of ILD. Otherwise, in cases in which ILD appeared more than 1 year after the onset of PH, patients were not included in our study.

Follow-up prospective investigations included World Health Organization (WHO) functional class, 6-min walk test, long-term supplemental oxygen, and RHC data. For the analysis, the follow-up visit used in the analysis was the first visit to include at least an RHC or a 6-min walk test within 1 year after initiation of the PAH-specific treatment.

Data collected at baseline included WHO functional class assessment, pulmonary function tests, 6-min walk test, RHC, and the presence of comorbidities.

### Data collection and proteomic analysis

#### Sample preparation

All sample preparation kits were purchased from ThermoFisher Scientific (Massachusetts, USA). First, albumin and immunoglobulins were removed from plasma samples with High Select HSA/Immunoglobulin Depletion Mini spin columns, and total protein concentrations were determined using Pierce BCA gold protein assay kit. Then proteins were treated and digested in each plasma sample using EasyPep kit.

#### Liquide chromatography mass spectrometry quantification

Thermo Scientific Ultimate 3000 RSLCnano system coupled with Orbitrap Q exactive High resolution mass spectrometer (Waltham, MA, USA) was used to qualitatively identify the peptides in plasma sample. Chromatographic separation was performed on a reversed-phase C18 column (300 μm × 15 cm, 1.9 μm) at nano-flow gradient (flow rate, 250 nL min^−1^). Peptides were pre-concentrated onto a PepMap 100 C18 column (5 µm, 100 Å, 0.3 mm × 5 mm). Then, they were separated onto a PepMap RSLC C18 column (2 µm, 100 Å, 75 µm × 500 mm). Water, acetonitrile (ACN), trifluoroacetic acid (TFA) and formic acid (FA) were purchased from Carlo Erba Reagents (Val-de-Reuil, France). Mobile phase A was water with 0.1% FA and mobile phase B was water with 80% ACN and 0.08% FA. The following gradient was applied: 0 3 min: 2.5% phase B; 3–47 min: from 2.5 to 25% phase B; 47–57 min: from 25 to 40% phase B; 57–60 min: from 40 to 90% phase B; 60–69 min: 90% phase B; 69–69.5 min: return to the initial condition of 2.5% phase B; 69.5–90 min: 2.5% phase B. For each sample, a volume of 1 µL was injected in the LC system. Peptides were detected and quantified by mass spectrometry in electrospray positive mode with a resolution of 70,000 in full scan. MS/MS analyses were performed by higher collision energy fragmentation (HCD) on the eight most abundant ions. The 10 most intense multicharged precursors were selected for collision dissociation analysis.

#### Identification of proteins and relative abundance

The analysis of the raw data files was performed using the Proteome Discoverer software (version 2.2.0.388, ThermoFisher), using label free quantification (LFQ) to compare group between survivors and non-survivors. Proteins were identified using an Uniprot reviewed database of Homo sapiens (http://www.uniprot.org/). Peptides used for protein quantification were «Unique + Razor». It means that peptides were not shared between different protein or that shared peptides were used only for quantification of proteins that have more identified peptides. Moreover, only peptides with at least 6 aminoacids and a high confidence rate in their identification were used to identify proteins. Then proteomic dataset was analyzed using Perseus software (https://maxquant.net/perseus/, version 1.6.15.0). After applying filters to avoid potential protein identification issues, values were log2 transformed. Then, proteins under a certain percent of 70% of valid in samples or identified by fewer than two unique peptides were eliminated. Finally, an imputation of the missing values was performed considering that proteins had a normal distribution.

### Statistical analysis

Statistical analysis was performed with the R software (URL https://www.R-project.org/), R Foundation for Statistical Computing, Vienna, Austria) for statistical computing and graphics.

Continuous variables are presented as median and interquartile range (IQR) Categorical variables are presented as numbers (percentages).

The primary aim was to seek for proteins associated with patients’ survival. Hence, the population study was divided into two groups, according to survival at 4 years:

- Short surviving patient defined by death within 1500 days of diagnosis
- Long surviving patients defined by survival to 1500 days

We chose an a priori cut-off of 1500 days to differentiate survivors from non-survivors, to be close to the median survival found in the literature [8]. In the survival analysis patients, were censored from the last visit. For the proteomic differential survival analysis, all censored patients lived longer than 1500 days. Data with p < 0.05 were considered as statistically different.

Comparison between patient samples for both analyses was performed using a two-sample Student's t-test. Multiple testing was taken into account by controlling the false discovery rate (FDR) at a level of 0.05 using Benjemini-Hochberg procedure. A multivariate selection procedure was additionally performed using a LASSO (least absolute shrinkage and selection operator) regression [21] with the glmnet R packages.

### Analysis of the proteins interactome

Interactions between regulated proteins in survivors and non survivors patients were evaluated on the String website (Search Tool for Retrieval of Interacting Genes/proteins, https://string-db.org/). This tool provides a database of known and predicted protein–protein interactions, including direct (physical) and indirect (functional) associations. These associations come from computational prediction, knowledge transfer between organisms and from other databases. We used the KEGG database to perform analyses of the biological processes in which the identified proteins were involved (https://www.genome.jp/kegg/pathway.html) [22].

## Results

### Study population

**Fig. 1:**
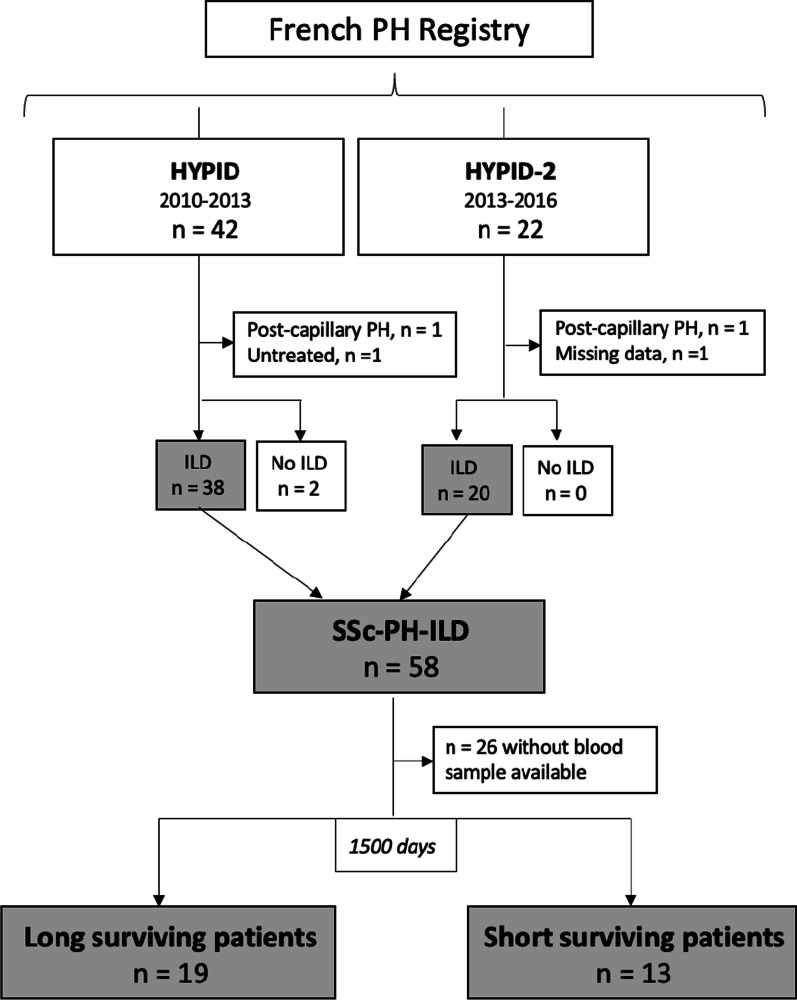
Study population. We have chosen an a priori cut-off of 1500 days to differentiate short surviving patients from long surviving patients, to be close to the median survival found in the literature

Among the 66 patients included in the HYPID study, 32 patients with systemic scleroderma associated with pulmonary hypertension and diffuse interstitial lung disease (SSc-PH-ILD) were eligible to the present study (Fig. 1).

### Overall survival

**Fig. 2:**
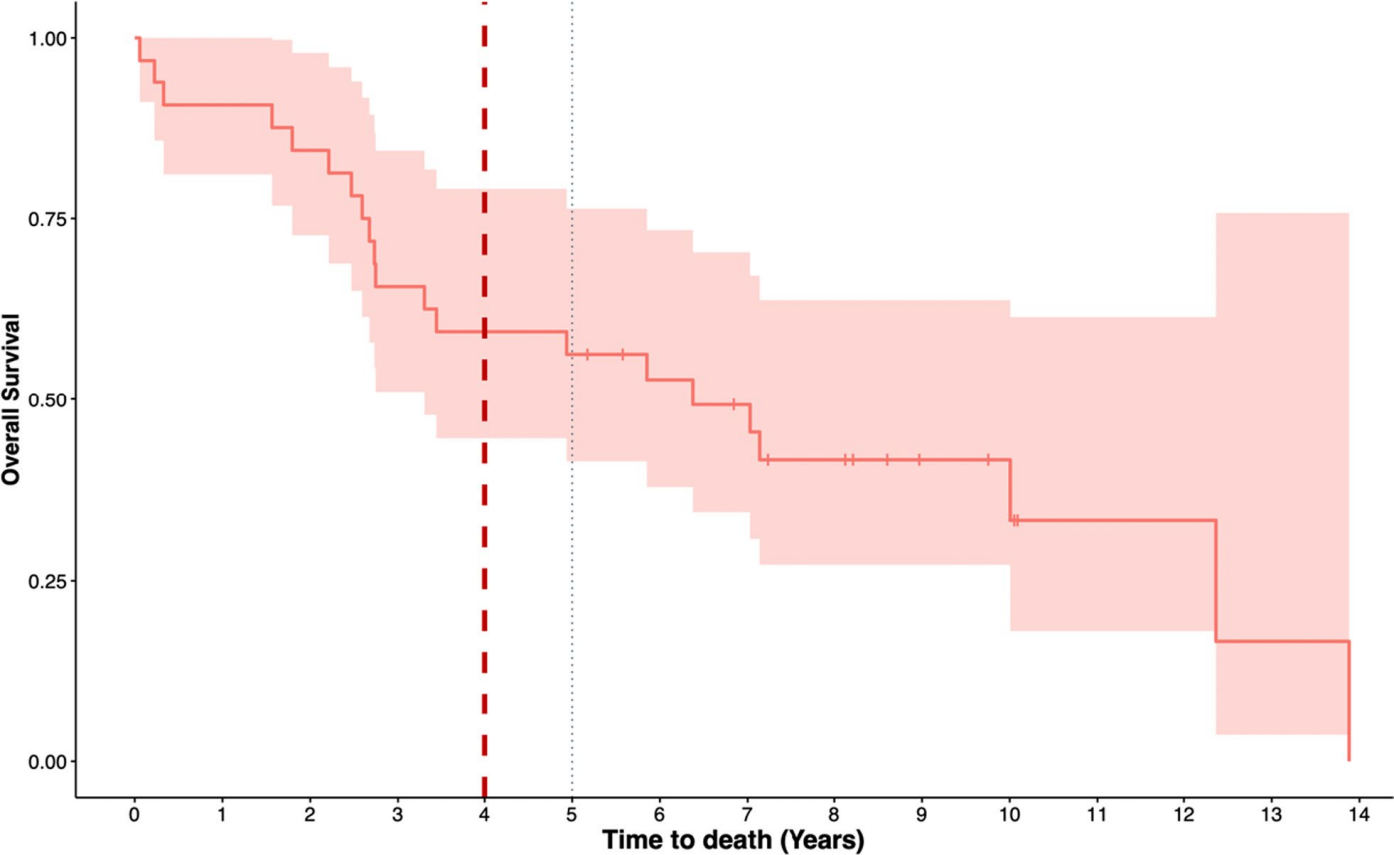
Survival plot for all patients. The red dotted line represents the 1500 days from diagnosis, allowing the distinction between patients who survived or not up to that date (“long surviving patients” and “short surviving patients” patients)

The median survival was 2327 days (6.3 years) (95% CI 1205-Na) and the crude 5-year mortality rate was 43.8% (95% CI 23.6–58.6%) (Fig. 2).

### Baseline characteristics

The baseline characteristics of long surviving patients and short surviving patients at day 1500 are presented in Table 1. At baseline, short surviving patients patients were more likely to be male, were younger and more likely to have limited cutaneous SSc than long surviving patients. Short surviving patients had more severe PH at baseline (first RHC) compared to long surviving patients, with higher mean PVR, higher mean PAP, and lower cardiac index. Compared to long surviving patients, short surviving patients had a lower carbon monoxide diffusing capacity, more severe hypoxaemia, and were more frequently on long-term supplemental oxygen therapy. Oxygen flow rate did not differ between groups in patients receiving long-term supplemental oxygen (median flow rate of 2 L/min).

**Table 1:** Baseline characteristics according to survival endpoint

	Long surviving patients *n* = *19*	Short surviving patients *n* = *13*
Demographic characteristics		
Age, median (IQR) years	60 (48.5–64)	51 (46.6–69.2)
Male gender, n (%)	4 (21)	5 (38.5)
BMI, kg/m^2^, median (IQR)	26.6 (23.8–31)	23.9 (22.1–27.1)
SSc cutaneous subtype, n (%)		
Limited	5 (27.7)	6 (50)
Diffuse	13 (72.2)	6 (50)
Autoantibodies, n (%)		
Anti-Scl-70	7 (36.8)	0 (0)
Anticentromere	0 (0)	2 (15.4)
Other/unknown	12 (63.2)	11 (84.6)
6-min walk test, median (IQR)		
Distance, meters	337 (298–416)	299 (225–476)
Borg dyspnea score	4 (3–5)	4 (1–5)
Biology, median (IQR) ^a^		
BNP (pg/mL) n = 5	64 (30–134)	57 (42–82.5)
Urate (µmol/L) n = 5	273 (208–328)	208 (207–227)
WHO functional class, n (%)		
1–2	7 (36.8)	1 (7.7)
3	10 (52.6)	10 (76.9)
4	2 (10.5)	2 (15.4)
Long term-term supplemental O2, n (%)	6 (31.6)	9 (69.2)
Comorbidities, n (%)		
Arterial hypertension	6 (31.6)	6 (46.1)
Diabetes	1 (5.3)	2 (15.4)
Coronary disease	0 (0)	0 (0)
Chronic kidney disease	0 (0)	0 (0)
Obesity	3 (15.8)	0 (0)
Hemodynamics, median (IQR)		
RAP, mmHg	7 (5.5–10)	6 (4–9)
Mean PAP, mmHg	30 (28.5–39.5)	38 (33–46)
Cardiac Index, liter/minute/m2	3 (2.7–3.5)	2,4 (2–2.8)
PVR, Wood units	4.4 (3–5.2)	6.2 (4.2–10.7)
PAWP, mmHg	9.5 (7–12)	12 (9–14)
Pulmonary function tests, median (IQR)		
FVC, % of predicted	62 (58–83.5)	62.5 (53–81)
FEV1, % of predicted	64 (51–76)	69.5 (57–75.5)
TLC, % of predicted	67 (59.76.5)	68 (51.7–78)
DLCO, % predicted	39 (35–44.2)	25 (21.7–30.5)
DLCO/VA, % predicted	63 (49.2–77)	44.5 (39.5–51.7)
PaO2 (mmHg) room air	81 (67–83.3)	54 (51–70.5)
PaCO2 (mmHg) room air	38 (34.9–42)	37 (34.5–41)
Radiological ILD pattern, n (%)^a^		
Nonspecific interstitial pneumonia	5 (45)	6 (50)
Usual interstitial pneumonia	6 (55)	2 (16.7)
Undetermined pattern	0 (0)	4 (33.3)
Extent of ILD on CT, n (%)^a^		
Limited	3 (25)	3 (25)
Extensive	9 (75)	9 (75)
%Extent of ILD on CT, median (IQR) ^a^	52.5 (28.7–70)	47.5 (27.5–66.2)
First-line PH treatment, n (%)		
ERA	17 (89.5)	10 (76.9)
PDE5i	1 (5.3)	2 (15.4)
ERA + PDE5i	1 (5.3)	0 (0)
Prostacyclin analogs + ERA + PDE5i	0 (0)	1 (8.7)
Sequential oral combination PH therapy during follow-up, n (%)	3 (15.8)	4 (30.8)
Other treatments, n (%)		
Diuretics	8 (42.1)	7 (54.8)
Oral anticoagulation	6 (31.6)	4 (30.8)
ILD treatment at PH diagnosis, n (%)		
None	4 (21)	7 (53.8)
Glucocorticoids alone	7 (36.8)	3 (23.1)
AZA	1 (5.3)	0 (0)
MMF	1 (5.3)	1 (7.7)
MMF + CYC	1 (5.3)	1 (7.7)
Missing	5 (26.3)	1 (7.7)
Duration of follow-up, median (IQR) days	2967 (2412–3606)	901 (571–997)

Except where indicated otherwise, values are the number (%) of patients.

*SSc–PH-ILD* systemic sclerosis with both pulmonary hypertension and interstitial lung disease, *IQR* interquartile range, *PAP* pulmonary artery pressure, *ERA* endothelin receptor antagonist, *PDE5i* phosphodiesterase 5 inhibitor, *AZA* azathioprine, *CYC* cyclophosphamide, *MMF* mycophenolate mofetil, *RTX* rituximab, *DLCO* diffusing capacity for carbon monoxide, *DLCO/VA* diffusing capacity for carbon monoxide divided by the alveolar volume, *PaO2* partial pressure of oxygen in arterial blood, *PaCO2* partial pressure of carbon dioxide, *FVC* forced vital capacity, *FEV1* forced expiratory volume in 1 s, *TLC* total lung capacity

^a^For some categories, data were not available for all 32 patients, as follows: n = 18 for BNP, n = 14 for urate, n = 30 for SSc subtype, n = 23 for Radiological pattern ILD, n = 24 for Percentage extension of interstitial damage, n = 14 for Extent of interstitial damage

There was a difference in dyspnea at baseline, with a higher proportion of WHO functional class I-II in long surviving patients, and a higher proportion of functional class III and IV in short surviving patients. There was no statistical difference between groups in 6-min walk distance. Both groups received diuretic and anticoagulant therapy in similar proportions.

The most frequent chest CT pattern observed among long surviving patients was usual interstitial pneumonia (55%), whereas among short surviving patients, the most commonly observed pattern was nonspecific interstitial pneumonia (50%). ILD was extensive in 75% of cases in both groups.

### Proteomic analysis

**Fig. 3:**
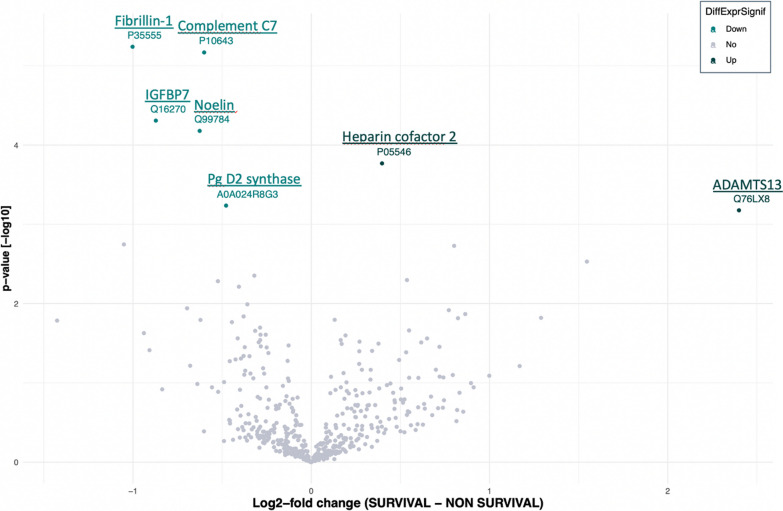
Volcano Plot of statistical significance against log2-fold change between long surviving patients vs short surviving patients at 1500 days at diagnosis

The combined analysis of the spectrometric data revealed a matrix comprising 1195 proteins (Additional file 1). After applying various filters, this number was reduced to 645 validated proteins. Statistical analysis evidenced significant changes in the expression (LFQ intensities) of 7 proteins between the two groups (long surviving patients or short surviving patients). Survival status was associated with increased levels of two proteins and decreased levels of five proteins (Fig. 3).

Proteins increased in long surviving patients were thrombospondin motifs 13 (ADAMTS13) and Heparin cofactor 2, belonging to disintegrin and metalloproteinase families of proteins, respectively.

**Fig. 4:**
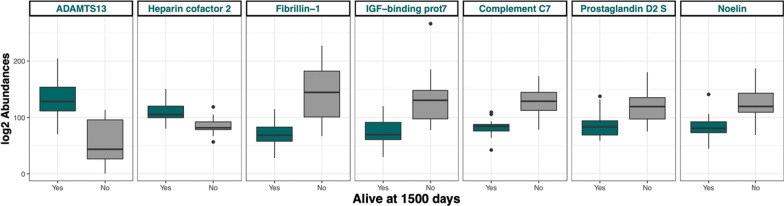
Boxplot showing the relative abundance of selected proteins in long surviving patients (green box) vs short surviving patients (grey box) 1500 days after the diagnosis

Proteins decreased in long surviving patients were Prostaglandin D2 synthase, Noelin, Complement component C7, insulin-like growth factor-binding protein 7 and Fibrillin-1 (Table 2 and Fig. 4).

**Table 2:** List of protein differentially regulated in long surviving patients versus short surviving patients 1500 days after the diagnosis

Protein Accession numbers	Protein names	Gene names	Adjusted p-value
Q76LX8	A disintegrin and metalloproteinase with thrombospondin motifs 13	ADAMTS13	0.048
P05546	Heparin cofactor 2	SERPIND1	0.017
A0A024R8G3	Glutathione-independent PGD synthase	PTGDS	0.048
Q99784	Noelin	OLFM1	0.008
P10643	Complement component C7	C7	0.002
Q16270	Insulin-like growth factor-binding protein 7	IGFBP7	0.008
P35555	Fibrillin-1	FBN1	0.002

**Fig. 5:**
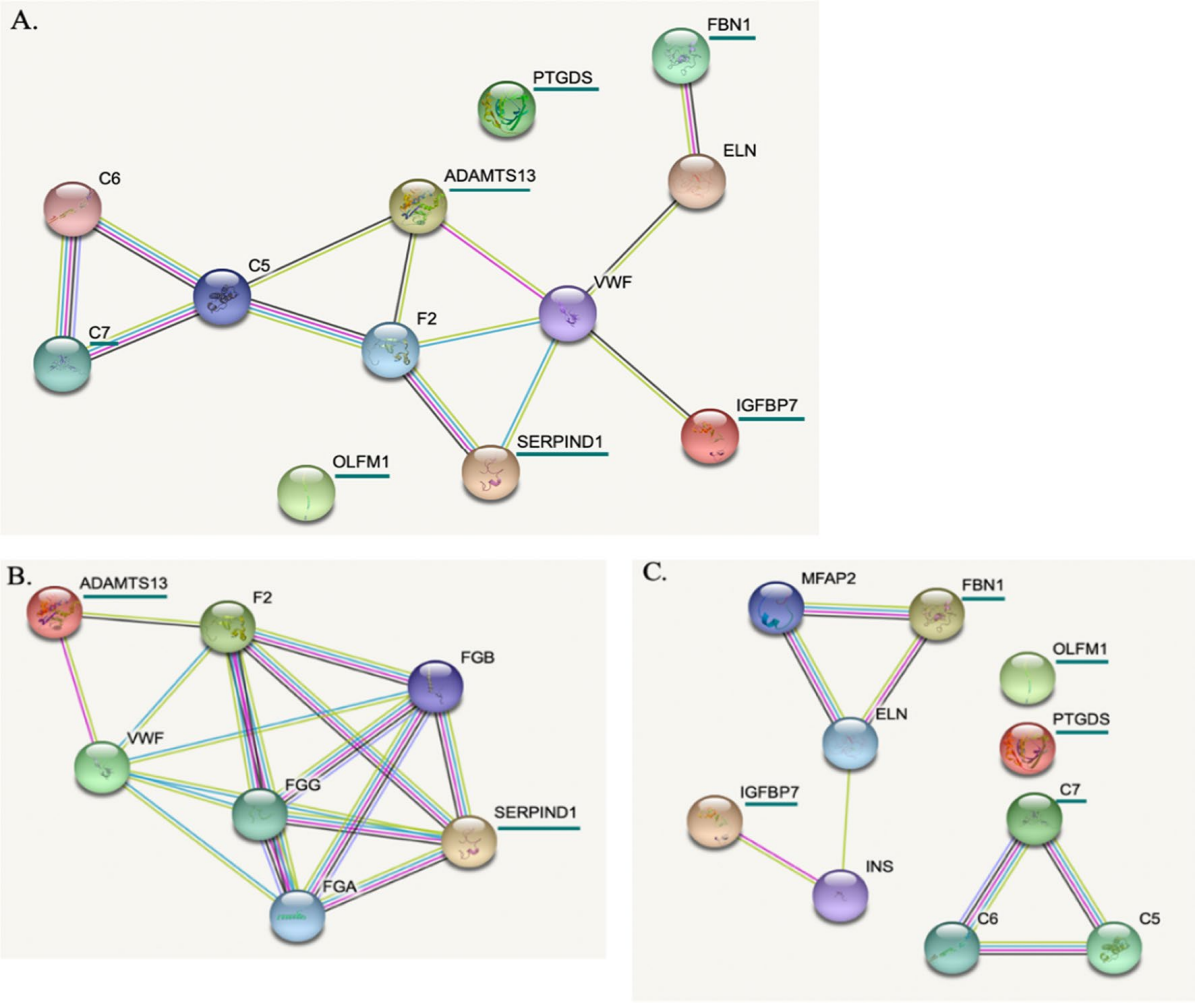
The protein–protein interaction network obtained using String software. In order to highlight the different links between the 7 significantly identified proteins, we performed an enrichment by adding “intermediate proteins” that link identified factors. Proteins of interest are underlined in green. **A** The protein-protein interaction network for the 7 identified significant proteins after enrichment by adding “intermediate connecting proteins”. **B** The protein-protein interaction network for the 2 identified significant proteins increased in long surviving patients compared to short surviving patients after enrichment by adding intermediate proteins **C.** The protein-protein interaction network for the 5 identified significant proteins increased in short surviving patients compared to long surviving patients after enrichment by adding intermediate proteins. *ADAMTS13* A disintegrin and metalloproteinase with thrombospondin motifs 13, *SERPIND1* Heparin cofactor 2, *FBN1* Fibrillin-1, *OLFM1* Noelin, *IGFBP7* insulin-like growth factor-binding protein 7, *PTGDS* glutathione-independent PGD synthase, *C7* complement component C7, *F2* prothombin, *C5* complement C5, *VWF* Von Willebrand factor, *C6* complement component C6, *ELN* elastine, *FGG* fibrinogen gamma chain, *FGA* fibrinogen alpha chain, *FGB* fibrinogen beta chain, *MFAP2* microfibrillar-associated protein, *INS* insulin

**Fig. 6:**
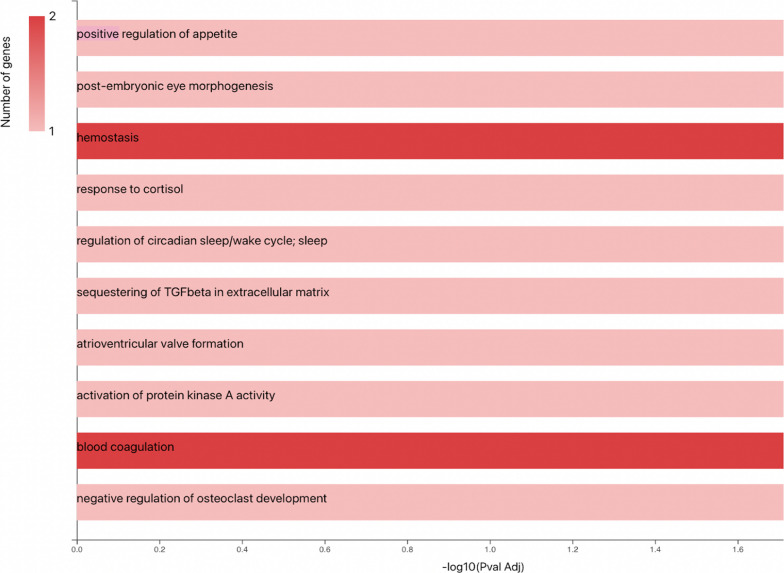
Bar Chart of the biological processes in which the identified proteins are involved

The potential interactions between proteins, whose expression is modulated by the survival criterion, were analyzed using the STRING software. As illustrated in the Fig. 5, except for the proteins PTGDS and OLFM1, the proteins could be easily connected to each other by relatively simple enrichment (addition of linker proteins) (Fig. 5A). In particular, the two proteins whose expression was decreased in short surviving patients (Heparin cofactor 2 and ADAMTS13) are linked via proteins involved in the blood coagulation process. This observation was further confirmed using additional analysis illustrating the biological processes involving these factors (gene ontology, Fig. 6). The proteins with increased expression in short surviving patients also showed connections but with different clusters, suggesting a more complex regulation of biological processes that were not directly related to each other (Fig. 5C). This point could explain the diversity of the biological process that are identified in Fig. 6.

## Discussion

In a proteomic analysis of 32 patients with SSc-PH-ILD, all included in a prospective multicenter national cohort, we identified 7 plasma proteins associated with haemostasis and fibrosis, and differentially expressed according to patients’ survival. In the long surviving patients, two proteins were increased (ADAMTS13, SERPIND1) and five were decreased (PTGDS, OLFM1, C7, IGFBP7, FBN1) compared to the short surviving patients.

In patients with SSc, two primary mechanisms of PH can coexist: group 1 (SSc-PAH) and group 3 (SSc-PH-ILD). While specific treatment for PAH have shown effectiveness in group 1, their efficacy and safety in group 3 have been a subject of debate [23]. Furthermore, most of the patients in this study were treated with ERAs and in particular BOSENTAN, which may seem outdated at present. These observation may suggest a lack of comprehension regarding the mechanisms underlying group 3 PH, while also highlighting the heterogeneity of patients. Despite the promising outcomes obtained in PH-ILD from the phase 3 Randomized Control Trial (RCT) of inhaled treprostinil (INCREASE) [24], these two conditions have distinct prognostic implications and necessitate contrasting therapeutic approaches. Therefore, it is crucial to differentiate between them effectively. Nevertheless, even with careful clinical and radiological evaluation, the classification of PH may be represent challenges in several patients. Indeed, a mixed involvement could be suspected in any patient with an identified ILD, even if minimal. With this perspective in mind, we have chosen to adopt an inclusive approach by including all patients with SSc associated with PH and an ILD regardless of its extent. We hypothesized that the underlying biological processes are likely to be manifested as disturbance in circulating proteins, making proteomic analysis a viable option for unraveling prognosis.

By analyzing the biological processes associated with the identified proteins (using the KEGG database), we found that survival was associated with 7 proteins, involved in blood coagulation and fibrosis pathways.

Indeed, in the group of patients alive at 1500 days (“long surviving patients”), we observed an increase in two pathognomonic biomarkers of blood coagulation, namely ADAMTS13 and Heparin cofactor II. ADAMTS13 is a metalloproteinase present in circulation, responsible for cleaving ultra large vWF, which helps to maintain the balance between thrombosis and haemostasis [25]. In a recent study, patients with newly diagnosed PAH had lower plasma levels of ADAMTS13 than healthy controls, as well as those with PH due to heart failure and chronic thromboembolic PH (CTEPH) [26]. Another study found that ADAMTS13 levels were lower in CTEPH patients compared to healthy controls [27]. However, it is important to note that the plasma ADAMTS13 levels in these two studies were not compared to patients with PH associated with respiratory diseases. Nevertheless, based on these data and our own results, it appears that ADAMTS13 expression may be modulated in PH, and potentially associated with survival.

Heparin cofactor II (HCII) is a serine protease inhibitor (SERPIN) that acts to neutralize thrombin’s activity within subendothelial layer of the blood vessel wall, thereby influencing the blood coagulation cascade. HCII plays a protective role against vascular remodeling, which includes atherosclerosis, and plasma HCII activity could serve as a predictive biomarker and a novel therapeutic target for preventing cardiovascular disease [28]. However, to the best of our knowledge, no association between this protein and PH has been reported yet. These results suggest that haemostasis may be associated with survival either through the activation of primary haemostasis (ADAMTS 13) or coagulation (HCII), or through the interplay between haemostasis and the vascular endothelium.

In the group of short surviving patients, we observed an increase in 5 proteins, Prostaglandin D2 synthase (PGD2), Noelin, Complement component C7 (C7), insulin-like growth factor-binding protein 7 (IGFBP7) and Fibrillin-1 (FBN1). Interestingly, IGFBP7 and FBN1 upregulated in short surviving patients are recognized as a crucial factors in the pathogenesis of fibrotic diseases [29, 30]. Specifically, previous studies have shown a significant increase in IGFBP7 expression in the lung tissue of patients with fibrotic lungs condition such as Systemic Sclerosis-associated fibrotic lung disease (SSc-FP) and Idiopathic Pulmonary Fibrosis (IPF), but not in those with SSc-PAH [31]. In addition, FBN1 has been identified to inhibit endothelial cell proliferation while promoting apoptosis [29].

Taken together, these results suggest that IGFBP7 and FBN1 may reflect a more pronounced “fibrotic” phenotype and a lesser “vascular” phenotype in short surviving patients compared to long surviving patients SSc-PH-ILD patients.

The biological actions of prostaglandin D2 (PGD2) are associated with vasodilation, bronchoconstriction, platelet inhibition and inflammatory cells recruitment. A significant increase in PGD2 levels was observed in patients with right atrial dysfunction (RVD) compared to those without. In addition, they reported that an increase of L-PGDS suggest an RVD and that this may also constitute a major prognostic factor in predicting mortality in patients with pulmonary thromboembolism [32]. These finding are in accordance with the results of the INCREASE trial, which demonstrated the beneficial effects of an inhaled prostagladin agonist (Treprostinil) [24].

Complement component C7 is well-known for its central role in the innate and adaptive immune response, while Noelin is recognized for its regulation of axonal growth in the central nervous system. To date, it is difficult to formally link the pathophysiological application of these two proteins to this research theme.

This study has limitations, related to the biases inherent to ancillary studies. Mainly, despite access to a national multicenter cohort, the relatively small number of patients (n = 32) decreased the power of the statistical analysis. In addition, we have no further information on the course of diffuse interstitial lung disease, nor on the causes of death of the patients included in our study. These data could provide additional clues as to the distinction of phenotypes in these patients. However, proteomic analysis of plasma samples from PH patients continues to be a reliable method for identifying novel biomarkers associated with this disease as recently demonstrated in PAH [15]. The results of our exploratory study generate potential biomarkers associated with survival, in patients with SSc-PH-ILD and pave the way for further, larger-scale prospective studies.

## Conclusion

The prognosis of SSc-PH-ILD is dismal. By proteomic approach, we identified 7 proteins associated with survival. These proteins are involved in the haemostasis and fibrosis pathways. We therefore provide additional hypotheses on the complex role of coagulation, primary haemostasis and key players in the pathogenesis of fibrotic diseases in the pathophysiology of SSc-PH-ILD. If confirmed in future prospective studies, these biomarkers represent potential candidates to prognostic enrichment in future clinical trials.

### Supplementary Information

**Additional file 1: **Schematic representation of the biological processes involved in the 1195 proteins initially identified from the Cytoscape software.

## Data Availability

The data that support the findings of this study are openly available in the Pulmonary Hypertension in Interstitial Lung Disease (HYPID) and HYPID-2 consecutive observational studies (ClinicalTrials.gov identifiers: NCT01443598 and NCT02799771, respectively). The authors confirm that the data are available within the article of Chauvelot and al. [13].
