# Supplementary figures and images for: Proteomic biomarkers for survival in systemic sclerosis-associated pulmonary hypertension
Source: Respir Res. 2023 Nov 7;24:273. doi: 10.1186/s12931-023-02578-0 (PMC10629050; doi:10.1186/s12931-023-02578-0)

**Additional file**


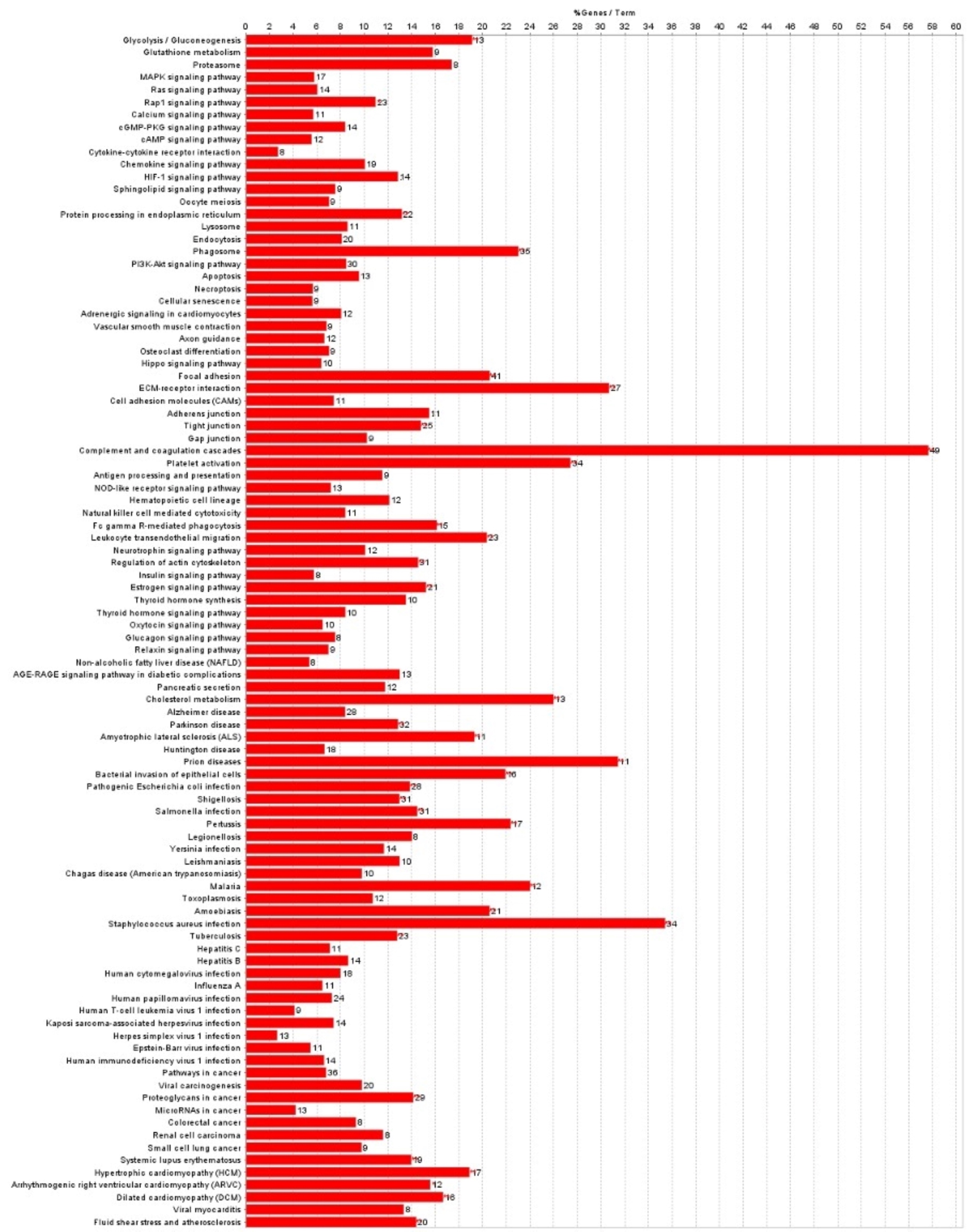

Supplement: Supplementary file 1 — Additional file 1: Schematic representation of the biological processes involved in the 1195 proteins initially identified from the Cytoscape software. [file 12931_2023_2578_MOESM1_ESM.docx]
